# Glutathione-Capped CdTe Quantum Dots Based Sensors for Detection of H_2_O_2_ and Enrofloxacin in Foods Samples

**DOI:** 10.3390/foods12010062

**Published:** 2022-12-22

**Authors:** Shijie Li, Linqing Nie, Lin Han, Wenjun Wen, Junping Wang, Shuo Wang

**Affiliations:** 1 Tianjin Key Laboratory of Food Science and Health, School of Medicine, Nankai University, Tianjin 300071, China; 2 Beijing Advanced Innovation Center for Soft Matter Science and Engineering, Beijing University of Chemical Technology, Beijing 100029, China; 3 State Key Laboratory for Food Nutrition and Safety, Tianjin University of Science and Technology, Tianjin 300457, China

**Keywords:** manganese dioxide nanosheet, water-soluble quantum dots with glutathione as ligand, fluorescence quenching immunosensors, antibiotic detection

## Abstract

Additives and antibiotic abuse during food production and processing are among the key factors affecting food safety. The efficient and rapid detection of hazardous substances in food is of crucial relevance to ensure food safety. In this study, a water-soluble quantum dot with glutathione as a ligand was synthesized as a fluorescent probe by hydrothermal method to achieve the detection and analysis of H_2_O_2_. The detection limits were 0.61 μM in water and 68 μM in milk. Meanwhile, it was used as a fluorescent donor probe and manganese dioxide nanosheets were used as a fluorescent acceptor probe in combination with an immunoassay platform to achieve the rapid detection and analysis of enrofloxacin (ENR) in a variety of foods with detection limits of 0.05–0.25 ng/mL in foods. The proposed systems provided new ideas for the construction of fluorescence sensors with high sensitivity.

## 1. Introduction

Food safety is directly related to people’s livelihoods, and it is also necessary to promote economic development and social harmony [1]. However, the globalization process of food trade has increased the risk of spreading contaminated food [2]. Food safety, as a global issue, has attracted increasing attention from governments, food industries and consumers. Food safety detection technologies play crucial roles in ensuring the health and safety of food for the population [3]. However, since most contaminants often exist in trace amounts, and complex food matrics seriously interfere with the detection results, the development of detection and analysis methods with higher sensitivity and accuracy has been the pursuit of food safety analysis [4]. For example, veterinary antibiotics such as enrofloxacin (ENR) are not completely biodegradable by animals, and their unfounded use can endanger human health in the form of prototypes or metabolites through the food chain [5,6,7]. In addition, H_2_O_2_ is fraudulently used to block microbial activity in milk that is near its sell-by date or unfit for consumption. As well, most countries have established maximum residue limits for veterinary drugs and hydrogen peroxide in food due to their toxic effects [8,9]. Therefore, it is essential to develop rapid and sensitive detection strategies for veterinary antibiotics and H_2_O_2_.

In the continuous innovation process of nanomaterials science, the detection technology based on fluorescent nanomaterials has gradually replaced the traditional detection and analysis methods based on large instruments due to the advantages of small instrument dependence, high signal sensitivity and short detection time, and has become a new direction for the development of food safety detection technology. The introduction of semiconductor quantum dots (QDs), up-conversion nanoparticles (UCNPs), atomic clusters (NCs), carbon dots (CDs) and other new fluorescent nanomaterials have promoted the development of fluorescent labeling technology, broadened the application range of fluorescent detection sensors [10,11,12,13]. QDs stand out among many fluorescent materials [14,15]. According to the different constituent elements and particle size, QDs achieve full coverage of the emission spectrum from the visible spectrum to the mid-infrared region [16,17]. Especially water-soluble QDs with biomolecules as ligands, due to their good biocompatibility, high quantum yield, and stable optical properties, have become a better choice for fluorescently labeled probes, and have a wide range of applications in fields of biochemistry, immunosensing, cell biology researches and so on [18,19]. Water-soluble QDs with GSH as a ligand (GSH-QDs) have a high degree of biocompatibility, mono-dispersity and stabilities [20,21,22]. The environmentally friendly GSH-QDs are easy to synthesize and easy to realize industrial production, which has promising commercial application prospects. In addition to its strong coordination ability, GSH also has a strong reducing ability, which can reduce MnO_2_ to Mn^2+^, and the -SH is oxidized to -S-S- to form GSSG [23]. Based on this, the redox reaction system between GSH and MnO_2_ is widely used in the field of biological monitoring, however, most reports still focused on the application of MnO_2_-based glutathione detection, and its contribution in the field of other target sensing analysis remains to be developed [24,25,26].

Nanosheets are a novel class of nanomaterials, which have been widely used in the fields of nanotechnology and nanomaterials [27]. Layered manganese dioxide nanosheets (MnO_2_ NSs) with high degrees of freedom are an important 2D layered functional material, which has attracted much attention due to its excellent optical properties and adsorption properties while retaining oxidative properties [28]. Based on the unique characteristics of GSH, water-soluble CdTe QDs with GSH as the ligand (GSH-CdTe QDs) were synthesized by hydrothermal method as the fluorescence signal probe. Firstly, an H_2_O_2_ detection method based on GSH-CdTe QDs was established for the highly sensitive detection of H_2_O_2_ in milk. In addition, a novel MnO_2_ NSs/GSH-CDTE QDs fluorescence quenching sensing system was constructed by coupling the detection antibody to the surface of MnO_2_ NSs as a sensing probe. Combined with the immunochromatographic analysis platform, a novel fluorescence quench immunosensor (FQISs) with strong anti-interference ability and high sensing sensitivity were constructed for the detection of ENR, and it is expected to be used for the detection and analysis of more trace hazards in food.

## 2. Materials and Methods

### 2.1. Chemicals and Materials

GSH, cadmium chloride hydrate (CdCl_2_), tellurium powder, sodium borohydride (NaBH_4_), NaOH, Bovine serum albumin (BSA), and ovalbumin (OVA), 1-(3-Dimethylaminopropyl)-3-ethylcarbodiimide hydrochloride (EDC), HEPES, NC membrane (Millipore HF90s) were purchased from Merck KGaA, (Darmstadt, Germany). MnO_2_ NSs were purchased from Nanjing XFNANO Materials Tech Co., Ltd. (Nanjing, China). PVC sheet, sample pad, conjugate pad and absorption pad were purchased from Shanghai Kinbio Tech.Co.,ltd (Shanghai, China), Enrofloxacin, flumequine, danofloxacin, sparfloxacin, gatifloxacin, fleroxacin, lomefloxacin, difloxacin, sarafloxacin, ciprofloxacin, and norfloxacin were purchased from Dr. Ehrenstorfer GmbH (Augsburg, Germany). A commercial ENR ELISA test kit was purchased from Reagen LLC (Moorestown, NJ, USA). Anti-ENR polyclonal antibody (Ab) was produced in our laboratory.

### 2.2. Synthesis of GSH-CdTe QDs

**Scheme 1:**
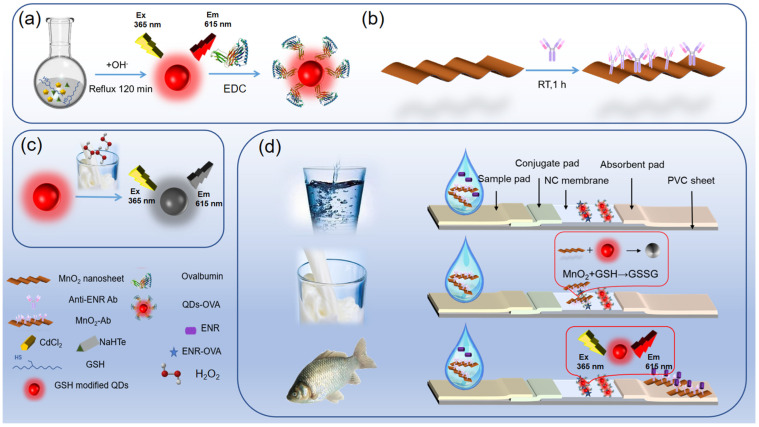
Schematic of FQISs. Preparation method of the GSH-CdTe QDs-OVA fluorescence probe (**a**) and MnO_2_-Ab fluorescence sensing probe (**b**), Detection procedures of FQISs (**c**) and schematic of fluorescence results under a UV lamp (**d**).

GSH-CdTe QDs with a final molar ratio of Cd^2+^/Te^2−^/GSH = 1:0.5:2.5 was synthesized by following a previous method with a minor modification [29]. A total of 0.25 mmol of Te powder and 1.32 mmol of NaBH_4_ were added into 3 mL of ultrapure water and stirred for 4 h under a nitrogen atmosphere in an ice bath to form the NaHTe precursor. At the same time, 0.5 mmol of CdCl_2_·5H_2_O and 1.25 mmol of GSH were dissolved into 120 mL of ultrapure water and adjusting the mixture to pH 10 with 1.0 M NaOH to form the cadmium precursor and kept stirred under bubbling nitrogen for 30 min. Then, the freshly NaHTe was added into the cadmium precursor immediately under a nitrogen atmosphere to form the CdTe QDs precursor. After refluxing for 2 h, the CdTe QDs with the maximum emission wavelength of 610 nm were prepared. Finally, the product was purified by centrifugation to remove free GSH and dispersed in ultrapure water and stored at 4 °C for use (Scheme 1).

### 2.3. Fluorescence Responses to H_2_O_2_ in Milk

1 mL of milk is dissolved in 9 mL of water, and without centrifugation and other pretreatments, 10 μL of the solution was mixed with 10 μL of GSH-CdTe QDs, diluted with water to 100 μL, and the fluorescence intensity of the solution was measured after standing for 10 min.

### 2.4. Fluorescence Responses to MnO_2_ NSs

100 μL of MnO_2_ NSs with different concentrations and 100 μL of 200 μg/mL CdTe QDs were added to each microwell in order, and the fluorescence value was immediately measured with a multifunctional fluorescent microplate reader and the fluorescent quenching rate (FQR) was calculated. FQR = (F_0_ − Fx)/F_0_ ∗ 100%, where F0 is the fluorescence value of QDs without adding MnO_2_ NSs, and Fx is the fluorescence value of QDs with MnO_2_ NSs added. In order to verify the cause of the fluorescence quenching phenomenon, the above CdTe QDs were replaced with ZnCdSe/ZnS QDs, CDs and RhB and the above experiment were repeated.

### 2.5. Preparation of Fluorescent Signal Probe (QDs-OVA)

OVA was coupled to the surface of GSH-CdTe QDs by chemical bonding to prepare a fluorescent signal probe. The detailed procedure was as follows: 10 μL of EDC (5 mg) and 10 μL of OVA (10 mg/mL) were added to 200 μL of GSH-CdTe QDs (200 μg/mL) with shaking incubation for 3 h at room temperature, then 10 μL of BSA (200 mg/mL) was added into the mixture and shaken for 10 min. The product was centrifuged to remove agglomerates, and the unconjugated protein was removed by 30 KDa ultrafiltration tubes, the conjugate was re-dissolved in 200 μL PBS and stored at 4 °C.

### 2.6. Preparation of Fluorescent Quenching Probe (MnO_2_ NSs-Ab)

The detection antibody of ENR (ENR-Ab) was coupled to MnO_2_ NSs by physical adsorption to prepare a sensor probe. A total of 1 mL of MnO_2_ NSs (50 μg/mL) was mixed with 2 μL of K_2_CO_3_ (0.2 mol/L) solution and 3 μL of ENR-Ab (0.65 mg/mL), and incubated for 1 h at room temperature. After that, 20 μL of 20% BSA solution and 10 μL of 10% PEG_20,000_ was added into the mixture and incubated for 30 min at room temperature, then the mixture was centrifuged at 12,000 rpm for 15 min and the precipitation was resolved into 200 μL of working solution.

### 2.7. Preparation of Enrofloxacin Coating Antigen (ENR-OVA)

ENR-OVA with a molar ratio of OVA to ENR of 1:25 was synthetized according to the mixed acid anhydride method [30] with slight modification; full details can be found in Supplementary Information (SI).

### 2.8. Detection Procedure

The details of the preparation of FQISs are provided in the Supplementary Information (SI). After preparing, 100 µL of standard or sample solution and 10 µL of the MnO_2_-Ab fluorescent quenching probe were mixed and dropped onto the sample pad with visual results being obtained within 10 min under a UV lamp. The detection principle is also provided in Supplementary Information (SI).

## 3. Results and Discussion

### 3.1. Characterization of GSH-CdTe QDs

**Figure 1:**
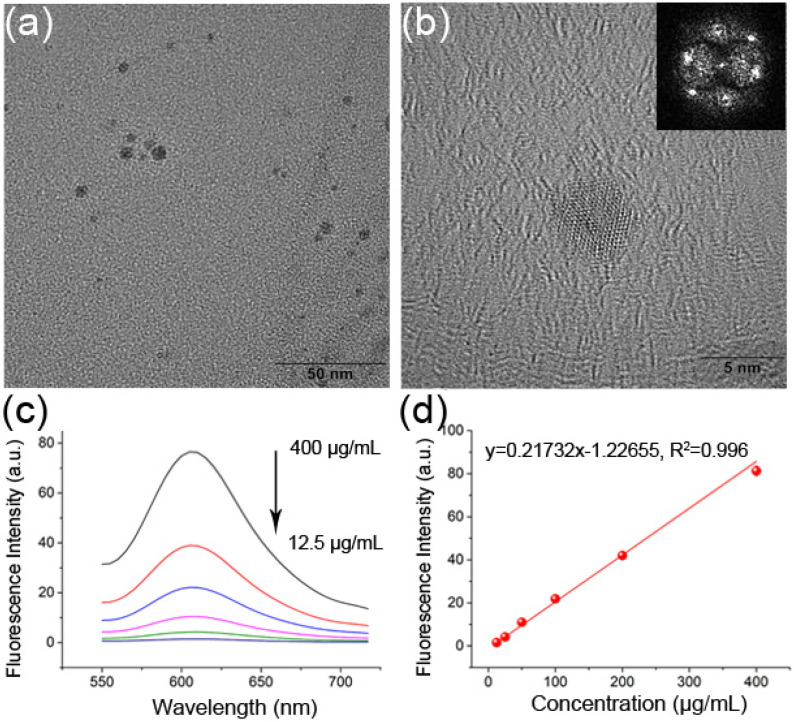
Characterization of GSH-CdTe QDs. TEM images (**a**) and HR-TEM image (Insert: Fourier diffractogram image) (**b**) of GSH-CdTe QDs. Fluorescence spectroscopy (**c**) and the linear relationship between emission fluorescence intensity at 615 nm (**d**) versus 12.5–400 μg/mL of GSH-CdTe QDs.

The synthesis procedure of GSH-CdTe QDs was shown in Scheme 1a, and transmission electron microscopy (TEM), high-resolution TEM (HR-TEM), UV-Vis absorption and fluorescence spectroscopy were used for the characterization of GSH-CdTe QDs. GSH-CdTe QDs with an average diameter of 3.2 nm (Figure S1) had a good dispersive crystal structure, and it was found that the lattice fringe (d = 0.23 nm) corresponded to the (200) crystal plane of CdTe blende (Figure 1a,b), which was consistent with the description in the previous report [31]. In addition, the optical properties of CdTe QDs were verified by fluorescence spectroscopy (Figure 1c,d). GSH-CdTe QDs had a concentration-dependent fluorescence emission peak at 615 nm under excitation at 365 nm, and its fluorescence intensity showed a steady linear decrease trend as the concentration of QDs decreased. The above results demonstrated the successful synthesis of QDs and their feasibility as signal components.

### 3.2. H_2_O_2_ Detection in Milk Powder

**Figure 2:**
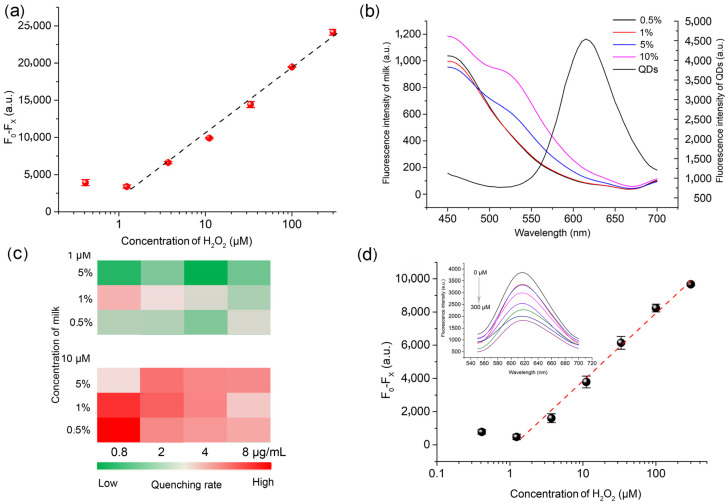
Detection of H_2_O_2_ by GSH-CdTe QDs in milk. Calibration curve for detection H_2_O_2_ in water (**a**); Fluorescence spectra of milk and GSH-CdTe QDs (**b**); Optimization results of milk and GSH-CdTe QDs concentration (**c**); And linear standard curve for H_2_O_2_ detection in Milk (Insert: Fluorescence spectra of GSH-CdTe QDs at each H_2_O_2_ concentration) (**d**).

The fluorescence of GSH-CdTe QDs was quenched in H_2_O_2_ (Figure S2), which was speculated to be caused by the oxidation of the QDs by the strongly oxidizing H_2_O_2_ leading to the generation of new defects on their surface, thus increasing the non-radiative transition, which in turn reduced the generation of fluorescence in the excitonic state, leading to fluorescence quenching [8]. It can be seen from Figure 2a that the fluorescence intensity gradually decreased with the increase of H_2_O_2_ concentration in a logarithmic trend rather than a linear trend, indicating that the quenching of the fluorescence of QDs was achieved through complex multivariate interactions. For the detection of H_2_O_2_ in water, the linear range was 1.23–300 μM of H_2_O_2_ with an LOD of 0.61 μM (S/N = 3). H_2_O_2_ is often used as a bactericide and other food processing aids in the production of milk and dairy products. However, milk has obvious fluorescence in the visible light range due to its complex composition. To verify the feasibility of GSH-CdTe QDs to detect H_2_O_2_ in milk, the interference of milk samples on QDs fluorescence was first evaluated (Figure 2b). The fluorescence intensity of milk decreased gradually in the range of 450–650 nm and was negligible at 615 nm, the maximum emission wavelength of GSH-CdTe QDs. Therefore, milk hardly interferes with the GSH-CdTe QDs fluorescence signal. In addition, the selectivity of the GSH-CdTe QDs was evaluated to exclude possible interferences in milk samples (Figure S3). The fluorescence values of GSH-CdTe QDs were reduced when the content of κ-casein and BSA was at 1%, but considering the large dilution factor of milk and milk powder in the assay, proteins such as κ-casein can not cause significant effects. Other than that, other interferents did not cause a decrease in the fluorescence of GSH-CdTe QDs. Therefore, the probe was considered suitable for the detection of H_2_O_2_ in milk. Furthermore, the checkerboard assay was used to evaluate the optimal operating conditions of GSH-CdTe QDs for the detection of H_2_O_2_ in milk (*n* = 3) (Figure 2c). The results showed that 1% milk solution containing 0.8 μg/mL GSH-CdTe QDs had the best detection efficiency and sensitivity. Under the above working conditions, the detection method of H_2_O_2_ in milk was constructed (Figure 2d), and the linear range was 1.23–300 μM of H_2_O_2_ with the LOD of 0.68 μM (S/N = 3).

Many colorimetric, fluorescent and electrochemical detection methods were developed for the detection of H_2_O_2_ based on peroxidase (or nanozyme) or using the strong oxidation of H_2_O_2_. Some representative approaches are summarized in Table 1. Hani et al. [32]. prepared Ce-MOF nanozyme, and constructed a fluorescence detection method for detecting H_2_O_2_ by the reaction of H_2_O_2_ with the Ce node of Ce-MOF. The detection range of H_2_O_2_ by this method was 200–1500 µM with an LOD of 10 µM. Wang et al. synthesized CeO QDs, and the introduction of QDs improved the charge transfer efficiency, thus enabling the electrochemical detection of H_2_O_2_. The method can detect and analyze H_2_O_2_ from 294 μM to 1.47 mM with an LOD of 26.5 µM [33]. These detection sensors achieve the quantitative detection of H_2_O_2_, but the process of probe preparation is complex, with harsh conditions and long preparation cycles. Zhang et al. established an H_2_O_2_ electrochemical detection platform based on Cat-HMFs/GCE, which achieved H_2_O_2_ detection from 100 μM to 3 mM with the LOD of 50 μM [34]. Biological enzymes have good catalytic activity, but the susceptibility of biological enzymes to deactivation limits their application. Thus, K.V. et al. cleverly designed an H_2_O_2_ detection paper chip by using the peroxidase property of chitosan. The linearity of the chitosan-based assay was found to be in the range of 10 μM to 10 mM with an LOD of 1.55 μM [35]. Compared with biological enzymes, chitosan has higher stability, easier storage and transportation, and a wider application range. In this work, a water-soluble detection probe was prepared by a relatively simple preparation method, and a more sensitive and rapid quantitative analysis of H_2_O_2_ was achieved without the introduction of other reagents. It has incomparable advantages in terms of detection sensitivity and ease of detection.

### 3.3. Characterization of MnO_2_ NSs

**Figure 3:**
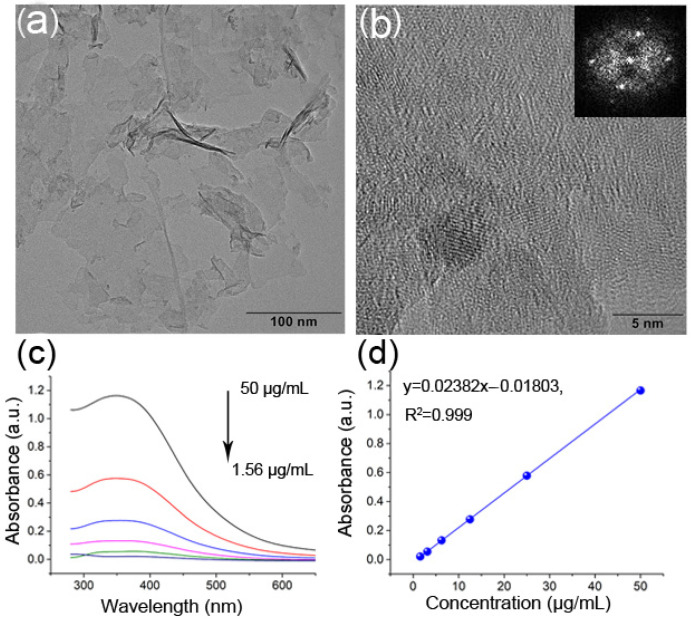
Characterization of MnO_2_ NSs. TEM images (**a**) and HR-TEM image (Insert: Fourier diffractogram image) (**b**) of MnO_2_ NSs; UV-Vis spectroscopy (**c**) and the linear relationship between Absorbance at 365 nm (**d**) versus 1.56–50 μg/mL of MnO_2_ NSs.

The redox reaction between GSH and MnO_2_ (MnO_2_ + 2GSH + 2H^+^➝Mn^2+^ + GSSG + 2H_2_O) is often used in biological applications. Inspired by this, MnO_2_ is intended to react with GSH, the ligand of GSH-CdTe QDs, to quench its fluorescence. Among the various forms of MnO_2_, MnO_2_ NSs have been considered as probe elements due to their good adsorption properties. Before verifying the fluorescence quenching ability of MnO_2_ NSs to GSH-CdTe QDs, the feasibility of MNO_2_ nanosheets as probe elements was first evaluated (Figure 3). The few-layered MnO_2_ nano-sheets with wrinkles and curling structures were observed by TEM image (Figure 3a,b), and it was found that the lattice fringe (d = 0.245 nm) corresponded to the (111) crystal plane of MnO_2_ [36]. In addition, the optical properties of MnO_2_ NSs were verified by UV-Vis absorption spectroscopy and fluorescence spectroscopy, respectively (Figure 3c,d), MnO_2_ NSs has an obvious concentration-dependent characteristic absorption peak at 350 nm, which was attributed to the result of d-d electronic transitions of manganese ions in the [MnO_6_] of layered MnO_2_ NSs. As well, its absorbance value decreased linearly with the decrease in MnO_2_ NSs concentration, which proved that the MnO_2_ NSs were homogenous and stable. The above optical data supplemented the characterization of MnO_2_ and also verified the feasibility of it as an optical signal component.

### 3.4. Feasibility Analysis of MnO_2_/GSH-CdTe QDs Fluorescent Sensing System

**Figure 4:**
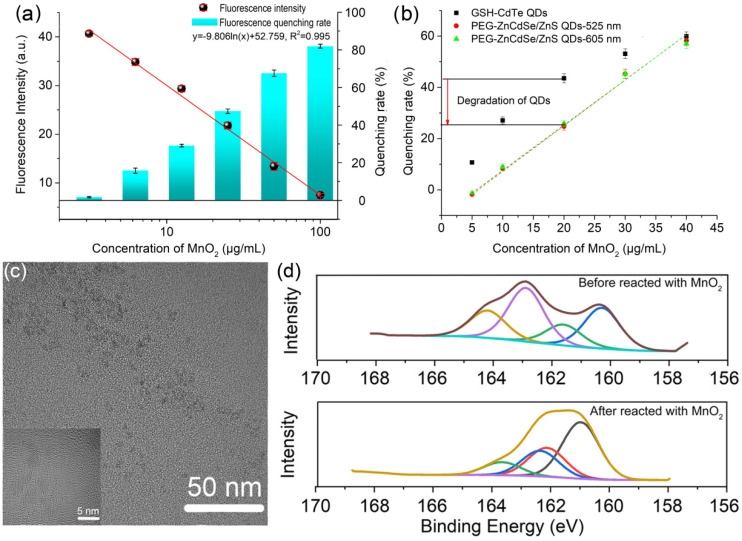
Verification of MnO_2_ NSs/GSH-QDs quenching system. Fluorescence intensity and quenching rate of GSH-CdTe QDs after reaction with different concentrations of MnO_2_ NSs (**a**); Comparison of the quenching ability of MnO_2_ NSs to deferent QDs (**b**); TEM images and HR-TEM images (Insert) of MnO_2_ NSs/GSH-QDs system (**c**); and XPS of GSH-CdTe QDs (S 2p) before and after reacted with MnO_2_ NSs (**d**).

Fortunately, the phenomenon of fluorescence quenching of GSH-CdTe QDs by MnO_2_ NSs was observed under UV light (Figure S2), and then the quenching performance was evaluated by fluorescence spectroscopy (Figure 4a). Similar to the quenching result of GSH-CdTe QDs by H_2_O_2_, the fluorescence value of GSH-CdTe QDs showed a logarithmic decrease trend rather than a linear trend with the increase of MnO_2_ NSs. This result tentatively demonstrated that MnO_2_ NSs and GSH-CdTe quantum dots can establish a fluorescence quenching system, and also proved that the system was not simply contributed by the fluorescence internal filtration effect (IFE). To verify this conclusion, two non-thiol ligand QDs were selected as fluorescent donor elements, and the results were shown in Figure 4b. The fluorescence of these two non-thiol ligands QDs both quenched as linear decrease trends with the increase of MnO_2_ NSs, which was attributed to the IFE. In addition, the quenching efficiency of MnO_2_ NSs for non-thiol capped QDs was much lower than that for GSH-CdTe QDs, when 10 μg/mL of MnO_2_ NSs were added to GSH-CdTe QDs and non-thiol capped QDs with the same fluorescence intensity, the quenching rate of GSH-CdTe QDs (27.08%) was 2.2-fold higher than that of non-thiol capped QDs (8.4%). It was guessed that in addition to the IFE, the destruction of GSH ligands by MnO_2_ NSs led to the disintegration of QDs, thereby increasing the quenching rate. The classic redox reaction between MnO_2_ and GSH provided support for the construction of a highly sensitive MnO_2_ NSs/GSH-CdTe QDs fluorescence quenching system.

In order to further verify the above conclusions, MnO_2_ NSs and GSH-CdTe QDs were mixed in water and their structures were characterized and evaluated by TEM and X-ray photoelectron spectroscopy (XPS). The TEM image of the mixture showed that the structures of MnO_2_ NSs and QDs were significantly disintegrated, and the original morphology could no longer be observed (Figure 4c). At the same time, the results of H_2_O_2_ interacting with GSH-CdTe QDs showed that under the action of H_2_O_2_, the fluorescence of QDs decreased significantly (Figure 4a and Figure S4), and a logarithmic proportional relationship was obtained between the fluorescence intensity and the concentration of H_2_O_2_, which was consistent to the quenching trend of MnO_2_/GSH-QDs quenching system. It further proved that the oxidation of sulfide (−2) was an important cause of fluorescence quenching of GSH-CdTe QDs [37]. The XPS results of GSH−CdTe QDs before and after reaction with MnO_2_ NSs also showed clear changes in the binding energies of S2p. Before oxidation by MnO_2_, the binding energies of S 2p (Figure 4d) at 161.2, 162.8 and 164.2 eV were ascribed to the sulfur in CdS, CdTexS_1−x_ and sulfide (−2), and after oxidation by MnO_2_, the binding energies of S 2p at 163.6 eV was appeared instead of 161.2, 162.8 and 164.2 eV, which ascribed to S_2_^2−^ [31]. These results indicated that oxidation of the sulfide(−2) in the shell of CdS or CdTexS_1−x_ by MnO_2_ NSs, leads to the passivation layer being broken and effectively quenching the fluorescence [38]. In general, MnO_2_ NSs and GSH-CdTe QDs have the ability to establish a fluorescence quenching system, and compared to most fluorescence quenching signals based solely on optical changes, this fluorescence quenching system had higher fluorescence quenching efficiency and specificity.

### 3.5. Establishment of Fluorescence Quenching Immunosensors (FQIS)

In addition to the above-mentioned optical properties, the surface adsorption properties of the sheet structure of MnO_2_ NSs and the chemical covalent binding properties of the GSH-CdTe QDs provided the basis for the construction of immuno-probes (Scheme 1a,b). Before establishing the method, the specificity of the selected ENR-Ab was analyzed by indirect competition ELISA (Table S1). ENR-Ab did not recognize other quinolone antibiotics at all, except for a 1.8% cross-reactivity to ciprofloxacin, demonstrating the good specificity of ENR-Ab. As well, it is in agreement with previous reports from our group [39,40,41]. Through physical adsorption, the ENR detection antibody was coupled with MnO_2_ NSs in a suitable pH environment to prepare the fluorescent receptor immuno-probe (MnO_2_-Ab). OVA was coupled with the carboxyl/am group on the surface of the GSH-CdTe QDs by chemical bonding to prepare fluorescent donor probes (QDs-OVA). It was worth mentioning that the type of buffer during the preparation of QDs-OVA greatly affected the performance of the probe (Figure S5), QDs-OVA coupled in H_2_O and PBS showed strong fluorescence, but the fluorescence intensity decreased significantly in HEPES and MES buffers, especially in MES buffers, the fluorescence of QDs was almost completely quenched (Figure S5A). In addition, the QDs-OVA conjugated in water and HEPES solution aggregated as a line on the NC membrane, while the conjugate prepared in ionic buffer (PBS) could not aggregate on the NC membrane, and only the conjugate prepared in H_2_O was not washed out by the buffer during the chromatography. The above results demonstrate that the QDs-OVA coupling was successful in H_2_O and could be successfully immobilized on the NC membrane. The fluorescence quenching immunosensors (FQISs) (Scheme 1c) were constructed based on the immunochromatography platform with QDs-OVA immobilizing on the surface of the NC membrane as the Control line (C line), and the mixture of QDs-OVA and ENR-antigen fixing under C line as Test line (T line). The details of the preparation and detection principle of FQISs were listed in Supplementary Information (SI).

**Figure 5:**
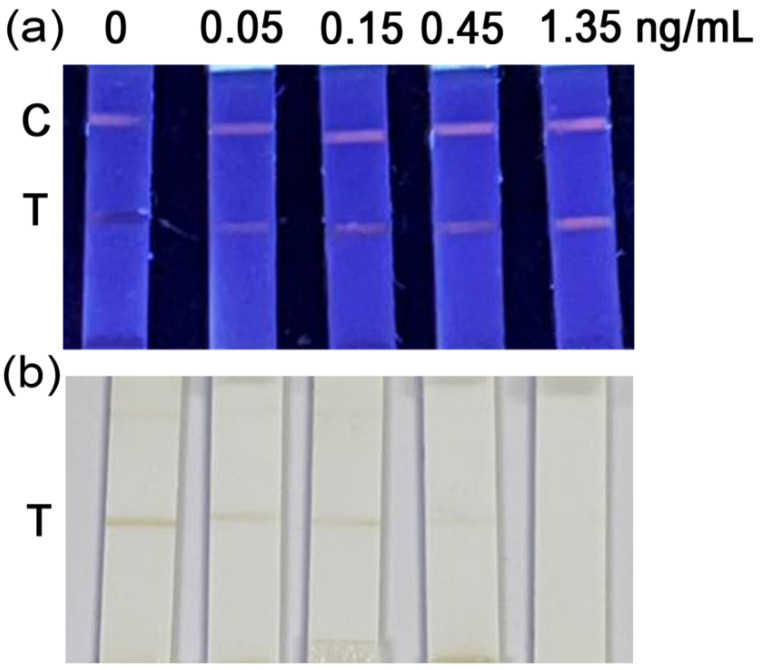
Detection of ENR using MnO_2_ NSs-based fluorescent quenching immunosensors (FQISs). A series of concentrations of ENR (0, 0.05, 0.15, 0.45 and 1.35 ng/mL) was prepared in pH 7.4 HEPES (20 mM) and detected under (**a**) UV lamp and (**b**) natural light.

During the working process of FQISs, the type of buffer was found to have significant impacts on the chromatography of MnO_2_ NSs (Figure S6). The MnO_2_-Ab probe aggregated in ionic buffers (PBS, PBST, NaCO_3_-NaHCO_3_ buffer) and could not be chromatographed on the NC membrane. However, it smoothly passed through the NC membrane and bound with the antigen at the T line in non-ionic buffers (MES, HEPES). It was guessed that the ionic solution destroyed the surface electronic environment of MnO_2_ NSs, leading to the aggregation of MnO_2_ NSs during the chromatography. The HEPES buffer (20 mM, Figure S7) was used for the establishment of FQISs, which was found to be more suitable for probe chromatography and antigen-antibody binding. Totals of 0, 0.05, 0.15, 0.45, and 1.35 ng/mL of ENR standards solution were mixed with 10 μL of MnO_2_-Ab probe and added to the sample pad, after 5 min of chromatography, the detection results were observed (Figure 5). Under a UV lamp (Figure 4a), the MnO_2_-Ab probe was enough for quenching the fluorescence of the T line when detecting ENR free solution. However, when detecting 0.05 ng/mL of ENR, some of the MnO_2_-Ab probes were bound by ENR, and the remaining probes were not enough to quench all the fluorescence at the T line, so the fluorescence appeared. Thus, the LOD of these FQISs was 0.05 ng/mL of ENR in the HEPES buffer. However, the brown strip on the T line disappeared under the sunlight until the concentration of ERN reached 1.35 ng/mL (Figure 4b).

Tap water, milk and crucian carp from different regions or brands (n = 3) were used for detecting to verify the practicability of FQISs (The details of sample preparation were shown in Supplementary Information (SI), and each sample was detected three times), and they were verified as ENR free samples by liquid chromatography-mass spectrometry. Totals of 0, 0.05, 0.1 and 0.5 ng/mL of ENR were added into the tap water, 0, 0.1, 0.2 and 0.5 ng/mL of ENR were added into milk samples and 0, 0.25, 0.5, and 1 ng/g of ENR were added to the crucian carp samples respectively, after mixing and standing overnight at 4 °C, the targets were extracted and detected by FQISs, and the detection results were shown in Table 2. There was no false positive detection result when detecting tap water and milk, but only one weak fluorescent strip (weaker than the LOD of FQIS) appeared when detecting one of the crucian carp samples in three consecutive tests. The ELISA kits also did not detect the presence of ENR in this sample, even after adding 0.25 ng/g of ENR. Indicating that the ENR in this crucian carp sample was less than 0.25 ng/g and the above results proved that the proper sample preparation process greatly reduced the influence of the matrix and avoided false positive results. The fluorescence appeared clearly at the T line when detecting water samples with 0.05 ng/mL ENR addition, milk samples with 0.1 ng/mL ENR addition and crucian carp samples with 0.25 ng/g ENR addition, thus the LOD of ENR by FQISs was 0.05 ng/mL in water, 0.1 ng/mL in milk and 0.25 ng/g in crucian carp. In addition, the above detection results were also confirmed by commercial ELISA kits to verify the accuracy of FQISs. The results showed that the detection results of FQISs agree with ELISA kits, and FQISs achieved faster detection with 2 to 10-fold higher sensitivity for food samples detection, respectively (Table S2).

Benefiting from the specific recognition performance of antigen-antibody, immunosensors are considered to be one kind of the most effective rapid detection and analysis tools. However, limited by the affinity of the antibody itself, the lower detection sensitivity of traditional immunoassays has not been improved. More and more functional materials are introduced into immunoassays as signal elements to improve sensitivity and other detection performance. Among them, the fluorescence signal has higher signal resolution efficiency than the colorimetric signal, and further, the fluorescence quenching signal performs better. In addition, differences in antibody loading rates and signal generation mechanisms of signal elements also have significant impacts on detection sensitivity. Reviewing the different immunochromatographic sensing systems established with the same detection antibody by our group, the above conclusions have been verified (Table 3).

The fluorescence system has made a contribution to improving sensitivity due to its stronger ability to resist background and matrix interference, thus the detection limit is decreased by 5-fold than the AuNPs system [40]. However, limited by lower antibody coupling efficiency and the “turn off” signal output mode, the sensitivity of fluorescence analysis needs to be further improved. Compared with the above two signal systems, the fluorescence quenching systems with “turn on” signal output mode for detecting small molecule targets take the appearance of fluorescence signal as the criterion of detection limit, so it has the highest detection sensitivity [40]. In the process of constructing fluorescence quenching systems, the matching of fluorescence donor and acceptor probes is the most important factor. Compared with AuNPs/PEG-QDs fluorescence quenching system, the higher fluorescence quenching efficiency of Ag nanoparticles (AgNPs) to CDs determines that the AgNPs/CDs quenching system has the ability to increase detection sensitivity. However, the blue fluorescence of CDs is similar to that of most biological samples or background light, so that the application of AgNPs/CDs quenching system in the detection of complex samples is limited.

It is worth mentioning that, compared with 0D nanomaterials, 2D nanomaterials show unique advantages in immunosensing analysis. For example, in the colorimetric system, 2D black phosphorus nanosheets (BPNSs) have better optical absorption and adsorption capacity than 0D gold nanoparticles (AuNPs). Therefore, the detection limit of immunosensors based on BP-Au nanocomposites is 10-fold lower than that of AuNPs-based immunosensors, and even lower than that of fluorescence detection systems [41]. In view of this, the organic combination of two-dimensional nanomaterials and fluorescence quenching signal system is bound to further improve the signal sensitivity. Two-dimensional MnO_2_ NSs were cleverly selected as fluorescent acceptor probes, and in addition to their excellent optical properties and adsorption properties, their chemical stability was stronger than that of BPNSs. More surprisingly, MnO_2_ NSs possessed unique and incomparable redox properties. Based on this, water-soluble GSH-CdTe QDs were selected as fluorescent donor probes, and a fluorescence quenching system of MnO_2_ NSs/GSH-CdTe QDs was constructed. In addition to fluorescence internal filtration, the irreversible disintegration of QDs due to the redox reaction between MnO_2_ and GSH further enhances the fluorescence quenching efficiency and specificity of this quenching system. Reassuringly, the novel fluorescence quenching system based on 2D MnO_2_ NSs brings amazing sensitivity for immunosensing assays. Compared with the AuNPs-based colorimetric detection results, the FQISs are 100-fold more sensitive when used for ENR detection, and approached the detection limit of photothermal quantitative detection. The construction of The FQISs lays the foundation for the in-depth study of the new fluorescence quenching system and its application in immunosensing, and is expected to promote the application of immunosensing analysis in the highly sensitive detection of trace pollutants. However, the work at this stage is still in the detection of antibiotic residues in primarily processed foods, and the current pre-treatment method is not yet able to achieve the extraction of antibiotics in deeply processed foods, and further exploration of the pre-treatment method for deeply processed food samples is the direction of subsequent research. In addition, the follow-up work will also actively advance this FQIS to a quantitative detection sensor to make it more accurate.

## 4. Conclusions

Water-soluble GSH-CdTe QDs with an average particle size of 3.2 nm and a maximum emission wavelength of 615 nm were successfully prepared by a solvothermal method. their H_2_O_2_ responsiveness was surprisingly found, and a highly sensitive and rapid label-free H_2_O_2_ fluorescence detection method was constructed based on it. When used for the detection of H_2_O_2_ in milk, the red fluorescence of GSH-CdTe QDs showed the advantage of being unaffected by the fluorescence of the milk matrix, which increased the detection accuracy and improved the detection sensitivity at the same time. The LOD was 0.61 μM of H_2_O_2_ in water and 68 μM of H_2_O_2_ in milk. In addition, the MnO_2_ NSs/GSH-CdTe QDs fluorescence quenching system was constructed. At 10 μg/mL of MnO_2_ NSs, the quenching rate of GSH-CdTe QDs was 2.2-fold higher than that of other ligand-capped QDs due to the oxidation of sulfide (−2) in the CdS or CdTexS_1−x_ shells by MnO_2_ NSs. Based on the MnO_2_ NSs/GSH-CdTe QDs fluorescence quenching system, we developed a highly sensitive fluorescence quenching immunosensor (FQIS). Targeting enrofloxacin, the FQISs achieved high sensitivity detection with a detection limit of 0.05 ng/mL in HEPES buffer, without the need for expensive detection equipment. For the detection of environmental water samples and food samples of animal origin, the results were consistent with commercially available ELISA kits. Benefiting from the MnO_2_ NSs/GSH-CdTe QDs fluorescence quenching system, the FQISs presented in this work have higher sensitivity than other quenching systems. Using water-soluble GSH-CdTe QDs as fluorescent probes, rapid and highly sensitive detection and analysis of contaminant residues such as additives and antibiotics in the environment and food was achieved. These promising strategies are expected to be used for the detection of other high-sensitivity biomolecules.

## Figures and Tables

**Table 1:** Review of methods for detection of H_2_O_2_.

Materials	Synthetic Conditions	Methods	Wavelength	LOD	Detection Range	Linear/Logarithmic Relationship	Ref.
Ce-MOF	Solvothermal, 85 °C for 16 h	Fluorescence	λex at 356 nm, λem at 540 nm	10 µM	200–1500 µM	Linear	[32]
CeO-QDs	Calcined at 900 °C for 3 h, stirring for 24 h in water and reacted at 120 °C for a further 6 h.	GCE	——	26.5 µM	294 μM–1.47 mM	Linear	[33]
Cat-HMFs	ultrasonication at 25 °C for 30 min	GCE	——	50 µM	100 μM–3 mM	Linear	[34]
Chitosan	Waterthermal, 60 °C for 30 min	Colorimetric	λ_Ab_ = 652 nm	1.55 µM	10 μM–10 mM	Logarithmic	[35]
QDs	Waterthermal, refluxing for 2 h	Fluorescence	λex at 365 nm, λem at 610 nm	0.61 µM	1.23–300 µM	Logarithmic	This work

**Table 2:** Comparison of the developed FISs and the commercial ELISA kit for the detection of ENR in tap water and crucian carp samples.

Sample	Conc. (ng/g(mL))	Detection Results (*n* = 3)	Observed Results	ELISA Kits (*n* = 3) (ng/g(mL))
Tap water	0	−; −; − ^a^	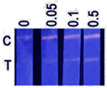	ND ^b^
	0.05	+; + ^c^; ± ^d^		ND
	0.1	+; +; +		ND
	0.5	+; +; +		0.47 ± 0.04
Milk	0	−; −; −	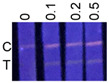	ND
	0.1	+; +; ±		ND
	0.2	+; +; +		ND
	0.5	+; +; +		0.42 ± 0.07
Crucian carp	0	−; −; ±	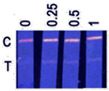	ND
	0.25	+; +; +		ND
	0.5	+; +; +		0.39 ± 0.08
	1	+; +; +		0.86 ± 0.11

^a^ ND: indicates no ENR detection or negative test result. ^b^ −: indicates a negative test result. ^c^+: indicates a positive test result ^d^ ±: indicates a weakly positive test result.

**Table 3:** An overview of immunosensors with the same antibody for determination of ENR.

Signal Type	Signal Element	Detection Limit (ng/mL)	Ref.
Colorimetric	AuNPs	5	[39]
	Black phosphorus-Au nanocomposite	0.5	[41]
Fluorescence	Dyed polymer microsphere	1	[39]
	PEG-QDs	1	[39]
Fluorescence quenching	AuNPs/PEG-QDs	0.25	[40]
	AgNPs/carbon dots(CDs)	0.1	[40]
Chemical degradation + fluorescence quenching	MnO_2_ NSs/GSH-QDs	0.05	This work

## Data Availability

The data presented in this study are available on request from the corresponding author.

## Supplementary Materials

The following supporting information can be downloaded at: https://www.mdpi.com/article/10.3390/foods12010062/s1, Methods: 1. Preparation of enrofloxacin coating antigen (ENR-OVA); 2. Preparation of FQISs; 3. Detection principle; 4. Sample preparation. Figures: Figure S1: Particle size distribution of GSH-CdTe QDs; Figure S2: Figure S2 Visualized results of GSH-CdTe QDs with or without MnO2 NSs under (A) Sun light and (B) UV light; Figure S3 The selectivity of GSH-CdTe QDs; Figure S4: Plots of fluorescence values versus H_2_O_2_ concentrations; Figure S5: Optimization of coupling conditions for fluorescent sensing probes; Figure S6: Chromatography results of fluorescent quenching probes in different buffers. From left to right: 20 mM of PBS, 30 mM of MES, NaCO_3_-NaHCO_3_ buffer, PBST, 20 mM of HEPES; Figure S7: Chromatography results of fluorescent quenching probes in different concentration of HEPES. From left to right: 10 mM, 20 mM, 50 mM and 100 mM of HEPES. Tables: Table S1 Specificity analysis of ENR-Ab, Table S2 Comparison of the analytical performance of the FQISs with the commercial ELISA test kit to detect enrofloxacin in animal origin food samples.
