# Supplementary material for: Glutathione-Capped CdTe Quantum Dots Based Sensors for Detection of H2O2 and Enrofloxacin in Foods Samples
Source: Foods. 2022 Dec 22;12(1):62. doi: 10.3390/foods12010062 (PMC9818724; doi:10.3390/foods12010062)
Supplement: Supplementary file 1 [file foods-12-00062-s001.zip › foods-1922905-supplementary.pdf]

Supplementary Materials

The Development of Immunosensors based on Two-Dimensional MnO_2 Nanosheet/Glutathione-Capped CdTe Quantum Dots High-Efficiency Fluorescence Quenching System

Shijie Li[1],Linqing Nie[1], Lin Han[2], Wenjun Wen[3], Junping Wang[3], Shuo Wang[1]**

[1]School of Medicine, Nankai University, Tianjin 300500, China

[2] Beijing Advanced Innovation Center for Soft Matter Science and Engineering, Beijing University of Chemical Technology, Beijing 100029, China.

[3]State Key Laboratory for Food Nutrition and Safety Tianjin University of Science and Technology, Tianjin, 300457, China

*Corresponding Authors:

E-mail: wangshuo@nankai.edu.cn

Fax: +86-22-85358445

Shijie Li and Linqing Nie：These authors contributed equally to this work.

Table of contents

Methods

1. Preparation of enrofloxacin coating antigen (ENR-OVA)

2. Preparation of FQISs

3. Detection principle

4. Sample preparation

Figures

Figure S1: Particle size distribution of GSH-CdTe QDs

Figure S2: Figure S2 Visualized results of GSH-CdTe QDs with or without MnO_2 NSs under (A) Sun light and (B) UV light.

Figure S3 The selectivity of GSH-CdTe QDs.

Figure S4: Plots of fluorescence values versus H_2O_2 concentrations

Figure S5: Optimization of coupling conditions for fluorescent sensing probes

Figure S6: Chromatography results of fluorescent quenching probes in different buffers. From left to right: 20 mM of PBS, 30 mM of MES, $NaCO_3$-$NaHCO_3$ buffer, PBST, 20 mM of HEPES

Figure S7: Chromatography results of fluorescent quenching probes in different concentration of HEPES. From left to right: 10 mM, 20 mM, 50 mM and 100 mM of HEPES.

Tables

Table S1 Specificity analysis of ENR-Ab

Table S2 Comparison of the analytical performance of the FQISs with the commercial ELISA test kit to detect enrofloxacin in animal origin food samples

1. Preparation of enrofloxacin coating antigen (ENR-OVA)

2.1 mg ENR dissolved in 0.3 mL of anhydrous DMF in an ice bath, 2 μL of tri-n-butylamine and 1.5 μL of isobutyl chloroformate were added drop by drop stirring. The mixture was stirred for 1 h at 4 °C in the dark. Next, product was dropped into 10 mg OVA dissolved in 1 mL carbonate buffer (0.05 mol/L, pH 9.6) and incubation for 12 h at 4 °C. The resulting coating antigen solution was dialyzed against PBS buffer (0.01 mol/L, pH 7.4) for 3 days at 4 °C.

2. Preparation of FQISs

The NC membrane was pasted onto the middle of the PVC sheet and overlapped 1 mm by a conjugate pad in left side and by an absorbent pad in right side. The QDs-OVA was immobilized at 0.7 μL/cm on the NC membrane as the control line (C line), and ENR-OVA was mixed with QDs-OVA and immobilized at 0.7 μL/cm at a distance of 0.5 cm to the C line as the test line (T line). Then, the membrane was dried at 37°C overnight in incubator. Finally, all pads were pasted onto a PVC sheet and cut into strips with widths of 0.35 cm.

3. Detection principle

The fluorescent quenching probe and sample solution was mixed and then added dropwise to the sample pad to start the detection procedure. Under the action of capillary force, MnO_2-Ab was chromatographed toward the absorbent pad, when detecting ENR free sample solution, the probe was bound by the coating antigen at the T line, and the fluorescence at the T line was quenched

under the action of MnO_2 NSs. However, when the ENR concentration in the sample solution was higher than the detection limit, the probe was bound by the target, resulting in less MnO_2 NSs at the T line, which was not enough to quench all fluorescence, and the fluorescence appeared. Regardless of whether the sample contains ENR or not, the C line did not bind any MnO_2 NSs and therefore has fluorescence, indicating that the detection was effective (Scheme 1D). Based on this principle, the fluorescence at T line appears from the quenched state and gradually became stronger with the increase of ENR concentration, so as to realize the rapid and visual detection of ENR. The detection limit (LOD) of the FQIS for ENR was defined as the concentration of ENR when fluorescence appears at the T line.

4. Sample analysis

For tap water samples, 1 mL sample was filtered with a 0.22 μm filter membrane, and 0.1 mL of 0.1 M PBS buffer was added for testing.

For milk samples, 1.0 g of milk was added into a centrifuge tube containing 1 mL of 3% trichloroacetic acid solution, the mixture was shaken and vortex for 5 min, and then centrifuged for 5 min (4°C, 10,000 rpm). The pH of supernatant was adjusted to neutral using 0.1 M NaOH and then diluted with equal amount of PBS for subsequent experiments. The dilution factor was 2.

For crucian carp samples, 2.0 g of edible tissue sample was transferred to a 50 mL centrifuge tube, 3.2 mL of acetonitrile and 0.8 mL of NaOH (0.1 M) were added and the tubes were vigorously shaken for 5 min. After

centrifugation at 5000 rpm for 5 min at room temperature, 2 mL of the supernatant was dried with nitrogen at 60°C. Then 1 mL of n-hexane and 5 mL of PBST buffer was added into the tube, and the mixture was centrifuged at 5000 rpm for 5 min at room temperature after shaken for 30 s, and the lower layer solution was analyzed using the FQISs. The dilution factor was 5.

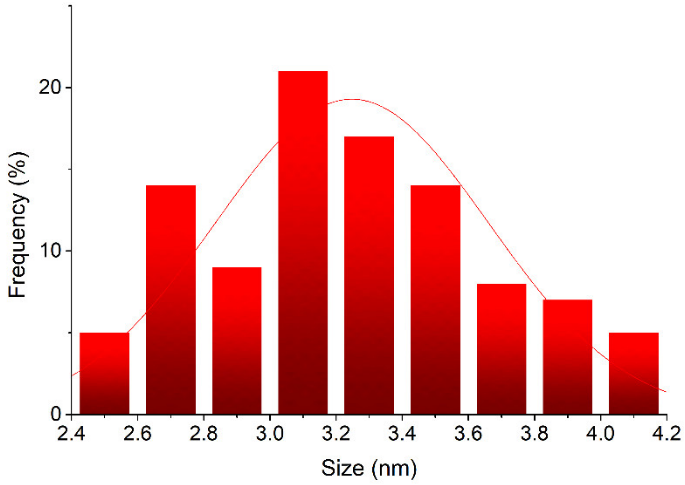

Figure S1 Particle size distribution of GSH-CdTe QDs

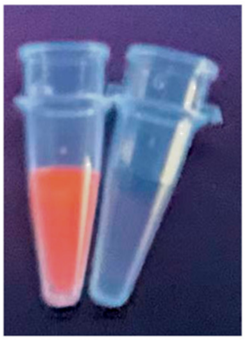

Figure S2 Visualized results of GSH-CdTe QDs with or without H_2O_2 under UV light.

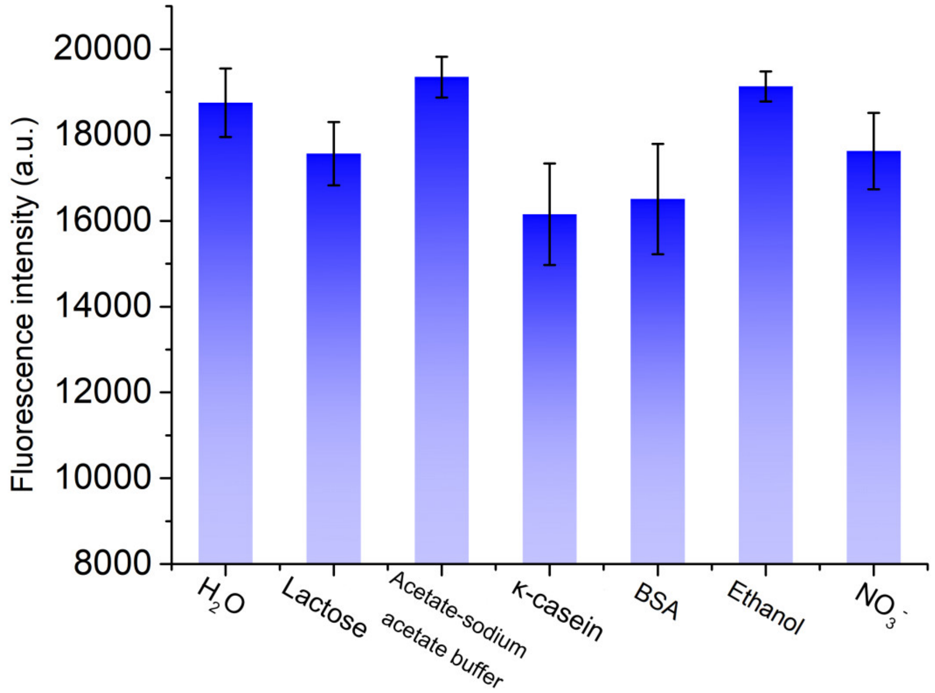

Figure S3 The selectivity of GSH-CdTe QDs. The concentration of lactose, κ-casein, BSA, ethanol and NO_3^- was 1%, and the pH of acetate-sodium acetate buffer was 5.5.

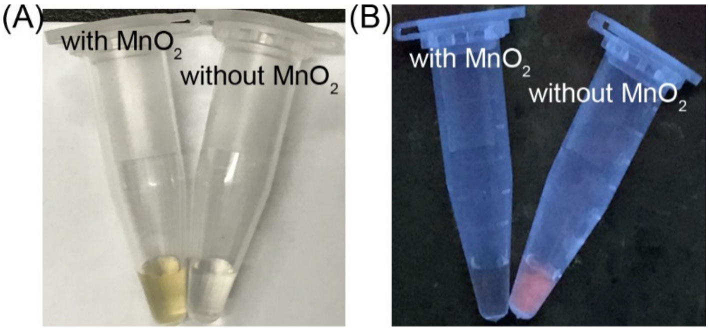

Figure S4 Visualized results of GSH-CdTe QDs with or without MnO_2 NSs under (A) Sun light and (B) UV light.

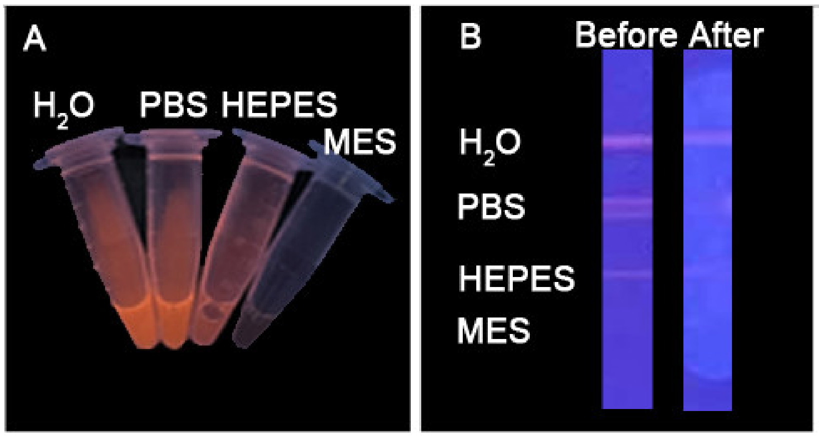

Figure S5 Optimization of coupling conditions for fluorescent sensing probes (A) Results of preparation of fluorescent sensing probes in different buffers; (B) Results of probes immobilized on NC membrane before and after buffer chromatography

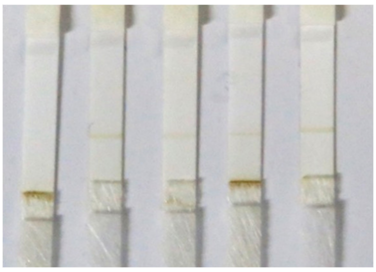

Figure S6 Chromatography results of fluorescent quenching probes in different buffers. From left to right: 20 mM of PBS, 30 mM of MES, $NaCO_3$-$NaHCO_3$ buffer, PBST, 20 mM of HEPES

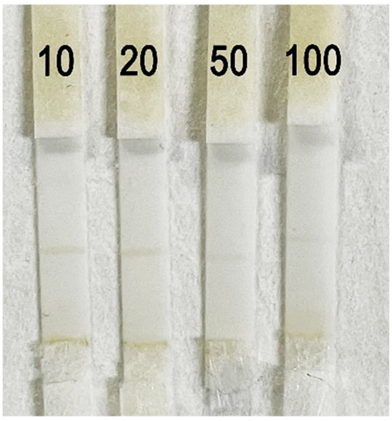

Figure S7 Chromatography results of fluorescent quenching probes in different concentration of HEPES. From left to right: 10 mM, 20 mM, 50 mM and 100 mM of HEPES.

Table S1 Specificity analysis of ENR-Ab

Target	Cross-reactivity rate
ENR	100%
Ciprofloxacin	1.8%
Danofloxacin	<0.1%
Sparfloxacin	<0.1%
Gatifloxacin	<0.1%
Flumequine	<0.1%
Norfloxacin	<0.1%
Fleroxacin	<0.1%

Table S2 Comparison of the analytical performance of the FQISs with the commercial ELISA test kit to detect enrofloxacin in animal origin food samples

	ELISA kits	FQISs
Sample test time	> 45 min	5 min
Result readout form	Reading absorbances under microplate reader	Visual inspection under portable UV lamp
Result measure time	5-10 min	On the spot
Detection limit	0.2 ng/mL (water)<br>0.4 ng/g (tissue)	0.05 ng/mL (water)<br>0.25 ng/g (tissue)
